# Supplementary material for: A pictorial account of the human embryonic heart between 3.5 and 8 weeks of development
Source: Commun Biol. 2022 Mar 11;5:226. doi: 10.1038/s42003-022-03153-x (PMC8917235; doi:10.1038/s42003-022-03153-x)
Supplement: Supplementary file 2 — Supplementary Information [file 42003_2022_3153_MOESM2_ESM.pdf]

A pictorial account of the human embryonic heart between 3.5 and 8 weeks of development. Hikspoors et al. 2021

Supplemental Table 1. Timeline of the appearance of embryonic heart structures and their adult terminology. The early heart grows by the serial addition of cells from the caudal and cranial second heart field. The first structure to develop is the embryonic left ventricle, which forms in late CS9 or early CS10 embryos. Subsequently, the common atrium and embryonic right ventricle form in late CS10 or early CS11 embryos. Myocardial expansion of the early heart is completed at CS11 and CS12, when the myocardial outflow tract and the venous sinus are added. The non-myocardial arterial component of the heart forms at CS15, when the aortopulmonary septum divides the aortic sac into the ascending aorta and pulmonary trunk. (hyperlink: 10.6084/m9.figshare.19102571)

structure	tissue	appearance	boundaries	alternate names	adult name
venous sinus	myocardial	CS12	systemic veins	sinus horns	coronary & systemic venous sinus; sinus node
			venous valves		
R & L atriums	myocardial	CS10-11			
			atrioventricular canal		
embryonic LV	myocardial	CS9			left ventricle
			interventricular foramen		
embryonic RV	myocardial	CS10			right ventricle
			narrowing lumen & transition trabeculations-to-endocardial ridges		
proximal OFT	myocardial	CS11		prox. myocardial OFT	infundibulum (R) & aortic vestibule (L)
			dog-leg bend		
middle OFT	myocardial	CS12		distal myocardial OFT	L & R arterial roots
			distal boundary myocard		
distal OFT	non-myocardial	CS15		ascending aorta & pulmonary trunk	intrapericardial arterial trunks
			pericardial reflection		
not part of heart	non-myocardial	CS15		aortic arch & brachiocephalic trunk	extrapericardial arterial trunks

A pictorial account of the human embryonic heart between 3.5 and 8 weeks of development. Hikspoors et al. 2021

Supplemental Table 2. List of embryos in the Carnegie collection that were used to reconstruct the heart and prepare 3D-PDF files. The list includes the correlation between the Carnegie stage of development of an embryo and its estimated post-fertilization (pf) age in days (range; [1]), its crown-rump length (CRL), the average CRL of same-stage embryos in the collection that had been graded "good" or "excellent" [2], with standard deviation (SD) and number of embryos (N). (hyperlink: 10.6084/m9.figshare.17144156)

Carnegie stage	range days pf	reconstr. specimen	CRL (mm)	ave. CRL (mm)	SD	N
9	25-27	3709	1.7	1.6	0.2	5
10	28-30?	6330	2.8	2.2	0.8	11
11	28-30	6344	2.5	3.1	1	20
12	29-31	8943	3.9	3.8	0.6	21
13	30-33	836	4	4.9	0.7	23
14	33-35	6502	6.7	6.5	0.9	37
15	35-37	721	9	7.7	1.2	24
16	37-40	6517	10.5	9.6	1.5	37
17	39-42	6520	14.2	12.2	1.3	26
18	42-45	4430	14	14.9	1.4	33
19	45-47			19.8	1.6	22
20	47-50	462	20	20.7	1.4	15
21	49-52			22.9	1.6	17
22	52-55			25.6	1	15
23	53-58	9226	31	28.8	2.4	21

A pictorial account of the human embryonic heart between 3.5 and 8 weeks of development. Hikspoors et al. 2021

Supplemental Table 3. List of structures identified in each of the reconstructed embryos. In total 75 items representing 70 different structures were reconstructed. Bilateral structures are usually shown separately so as not to hinder views. Eight structures not belonging to the cardiovascular system served as topographical landmarks. Almost all structures were identified in ≥2 successive Carnegie stages of development. Reconstructed cardiovascular structures are arranged in the upstream-to-downstream order that is also used in the description of heart morphology according to the "sequential segmental analysis" protocol [3]. Because of limited space that is available to describe the structures in the model trees accompanying the reconstructions, the following abbreviations were used: AV: atrioventricular; card: cardinal; CCS: (ventricular) cardiac conduction system; curv: curvature; DMP: dorsal mesenchymal protrusion; HCC: hepatocardiac vein; L: left; LA: left atrium; LV: left ventricle; musc: muscular; NCCs: neural crest cells; non-adj: non-adjacent; OFT: outflow tract; PAAs: pharyngeal arch arteries; pulm: pulmonary; R: right; RA: right atrium; RV: right ventricle; SAN: sinuatrial node; subpulm: subpulmonary; Umb: umbilical; Vit: vitelline.

Click on link: Figshare 10.6084/m9.figshare.17121977

A pictorial account of the human embryonic heart between 3.5 and 8 weeks of development. Hikspoors et al. 2021

Supplemental Figures 1-12. Interactive 3D-PDFs of human hearts between 3.5 and 8 weeks of development. Watch instruction video about its functioning or read the following text: Download a 3D-PDF to enable the interactive options. The 3D-PDFs can be opened on any computer as long as the Adobe PDF or equivalent reader is installed. A 3D-PDF becomes activated by "clicking" with the mouse on the reconstruction. A toolbar appears at the top of the screen that includes the option "model tree". The model tree displays a material list of structures in the upper box, and preset viewing options (cameras) in the lower box. The sequence of items corresponds to that in Supplemental Table 3. The list of visible structures can be modified by marking or unmarking a structure. To manipulate the reconstruction, press the left mouse button to rotate it, the scroll button to zoom in or out, and the left and right mouse buttons simultaneously to move the embryo across the screen. A structure can be rendered transparent by selecting that option from the drop-down menu after selecting the structure with the right mouse button. To inspect a combination of structures, one is advised to build up the composition, beginning with a familiar component, such as a lumen, rather than deleting non-relevant structures one-by-one from a completely reconstructed specimen. The slicer button in the toolbar allows making cross sections. The plane of section can be adjusted with the offset and tilt options. The "loop wires" in Supplemental Figures 3-6, which are drawn through the center of the endocardial heart tube, emphasize the changing shape of the heart loop during CS10-13. The side length of the scale cubes is 200 μm. The preset views correspond to the images shown in Figures 1-10. Note that items that are visible in these views can be altered by marking or unmarking a structure in the model tree.

Click on this link Figshare 10.6084/m9.figshare.17033225 to watch video 'Instruction video interactive 3D-PDF'.

Click on links (see next pages) Supplemental Figures 1-12 Figshare to download and open each interactive 3D-PDF.

A pictorial account of the human embryonic heart between 3.5 and 8 weeks of development. Hikspoors et al. 2021

Supplemental Figure 1. **Figshare: 10.6084/m9.figshare.17033156** **CS9 embryo at ~26 days after fertilization**. The cardiac jelly, which is produced by endoderm and myocardial cells in the visceral layer of the pericardium [4], represents the boundary of the primary myocardium developing within in this visceral layer (*cf.* [5]).

Supplemental Figure 2. **Figshare: 10.6084/m9.figshare.17033189** **CS10 embryo at ~28 days after fertilization.** The cardiac lumen now resembles an hourglass with its neck at the junction between the embryonic left and right ventricles. When viewed from dorsal the continuity between the left- and right-sided myocardium indicates that the dorsal mesocardium has disappeared at this location so that one can pass from left to right on the dorsal side of the heart tube. Further note that the junction of both embryonic ventricles bends slightly leftward and ventrally (see "loop wire"), which shows that cardiac symmetry has broken and cardiac looping starts.

Supplemental Figure 3. **Figshare: 10.6084/m9.figshare.17033249** **CS11 embryo at ~29 days after fertilization.** Note that the common cardinal veins are still absent. Further note the topographic relation between the inflow tract and the contour of the cranial intestinal portal. The twisted loop of the heart lumen is visualized by the "loop wire".

Supplemental Figure 4. **Figshare: 10.6084/m9.figshare.17033252** **CS12 embryo at ~30 days after fertilization.** The "spikes" on the lumen of the embryonic left and right ventricles represent the endothelial ingressions that mark the beginning formation of the ventricular trabeculae. Also note that the loop-wire model of the heart tube now resembles two helices that connect in the right ventricle.

Supplemental Figure 5. **Figshare: 10.6084/m9.figshare.17033255** **CS13 embryo at ~32 days after fertilization.** The epicardium has spread over a large part of the surface of the heart, but we have reconstructed only the areas with a thick layer of epicardium in the grooves of the atrioventricular and interventricular junctions.

Supplemental Figure 6. **Figshare: 10.6084/m9.figshare.17085503** **CS14 embryo at ~34 days after fertilization.** Note that, from this stage onwards, we use the spinal ganglia as reference for segmental levels and that the first 4 somites do not form ganglia [6]. Further note that the parietal and septal outflow-tract ridges form within the cuff of endocardial jelly, which itself then becomes reduced to a very thin layer that was no longer reconstructed.

Supplemental Figure 7. Figshare: 10.6084/m9.figshare.17085506 CS15 embryo at ~36 days after fertilization. Note that this specimen suffers from venous congestion (see also main text and Supplemental Figure 15). Further note that the aortic trunk consists of the intrapericardial ascending aorta and the extrapericardial brachiocephalic trunk and aortic arch.

Supplemental Figure 8. Figshare: 10.6084/m9.figshare.17085509 CS16 embryo at ~38 days after fertilization. Note that the right atrioventricular junction derives from the left-sided atrioventricular canal, but is from now on depicted as a right-sided connection. Also note that the interventricular foramen and its surrounding ring bundle of developing ventricular conduction system still resemble the configuration seen at CS14, but the part surrounding the connection between the left ventricle and subaortic outflow tract has disappeared (hatched section). Note further that the parietal and septal outflow-tract ridges both have a darker proximal part and a lighter distal part that contributes to semilunar valve formation. In addition, the tissue forming the intercalated spurs produces not only the future ventral and dorsal semilunar leaflets of the arterial valves (lighter shade), but also the walls of the ascending aorta and pulmonary trunk (darker shade).

Supplemental Figure 9. Figshare: 10.6084/m9.figshare.17085512 CS17 embryo at ~40 days after fertilization. This stage shows very pronounced changes in the shape of the outflow tract and its constituent structures.

Supplemental Figure 10. Figshare: 10.6084/m9.figshare.17085515 CS18 embryo at ~43 days after fertilization. Note that there is only a single (left) coronary artery that passes through the myocardium of the distal outflow tract.

Supplemental Figure 11. Figshare: 10.6084/m9.figshare.17085521 CS20 embryo at ~49 days after fertilization. Note that the superior and inferior atrioventricular cushions are no longer distinguishable as separate entities and are, therefore, depicted by hatching both code colors.

Supplemental Figure 12. Figshare: 10.6084/m9.figshare.17085524 CS23 embryo at ~56 days after fertilization. Note that the periaortic section of the GlN ring is no longer reconstructed.

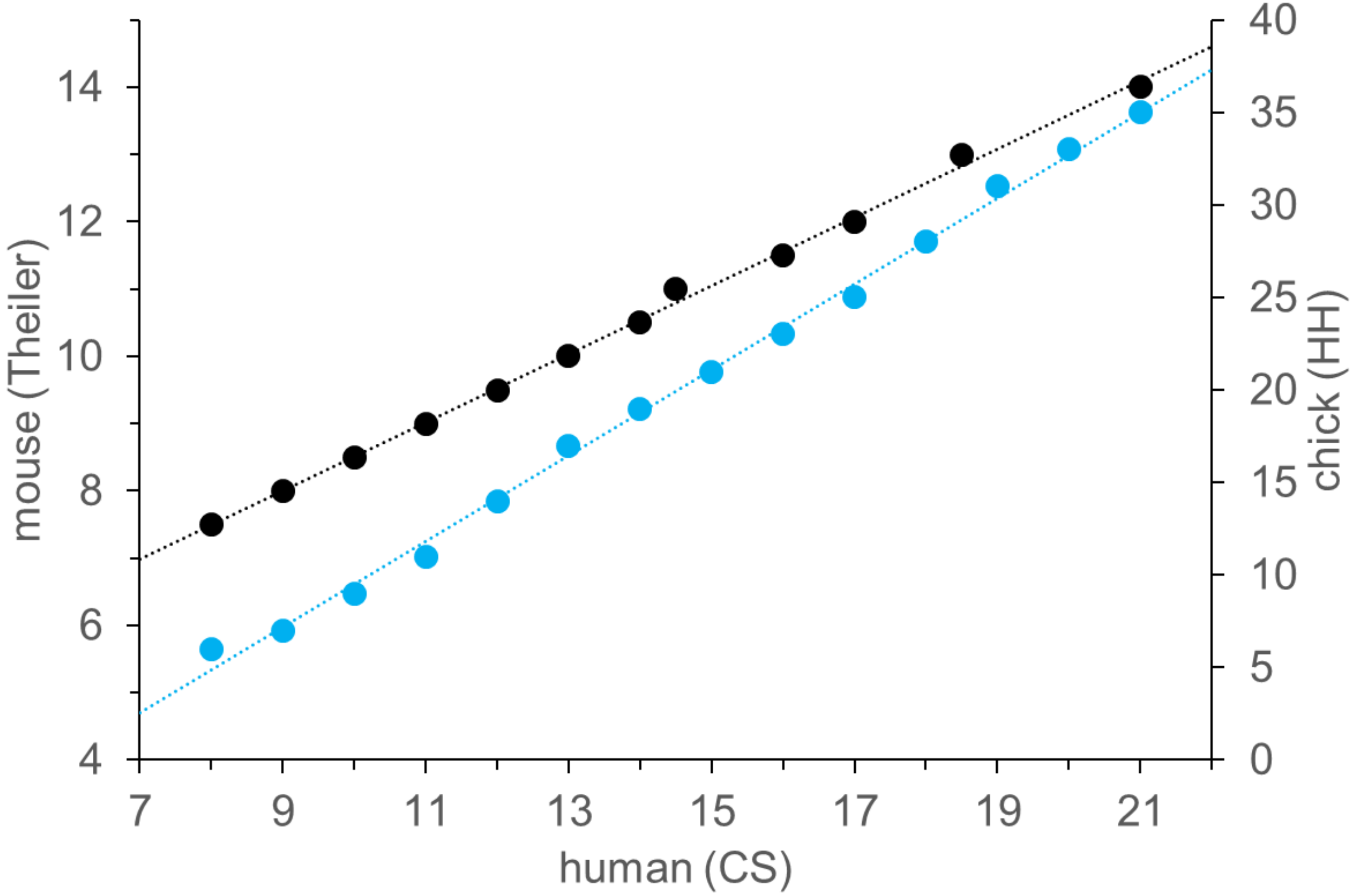

Supplemental Figure 13. Relation between developmental stages in men and mice, or chicken. Developmental stages in man (horizontal axis) are expressed in Carnegie Stages [1]; those in mice (left Y-axis) in days of embryonic development corresponding with Streeter's Horizons [7]; and those in chicken (right Y-axis) in Hamburger-Hamilton stages [8]. Black symbols relate development of men and mice, while blue symbols relate men and chickens. Although established staging systems for mouse embryos exist (e.g. that of Theiler [7]), they are rarely used in mouse developmental cardiology. (hyperlink: 10.6084/m9.figshare.17143910)

A pictorial account of the human embryonic heart between 3.5 and 8 weeks of development. Hikspoors et al. 2021

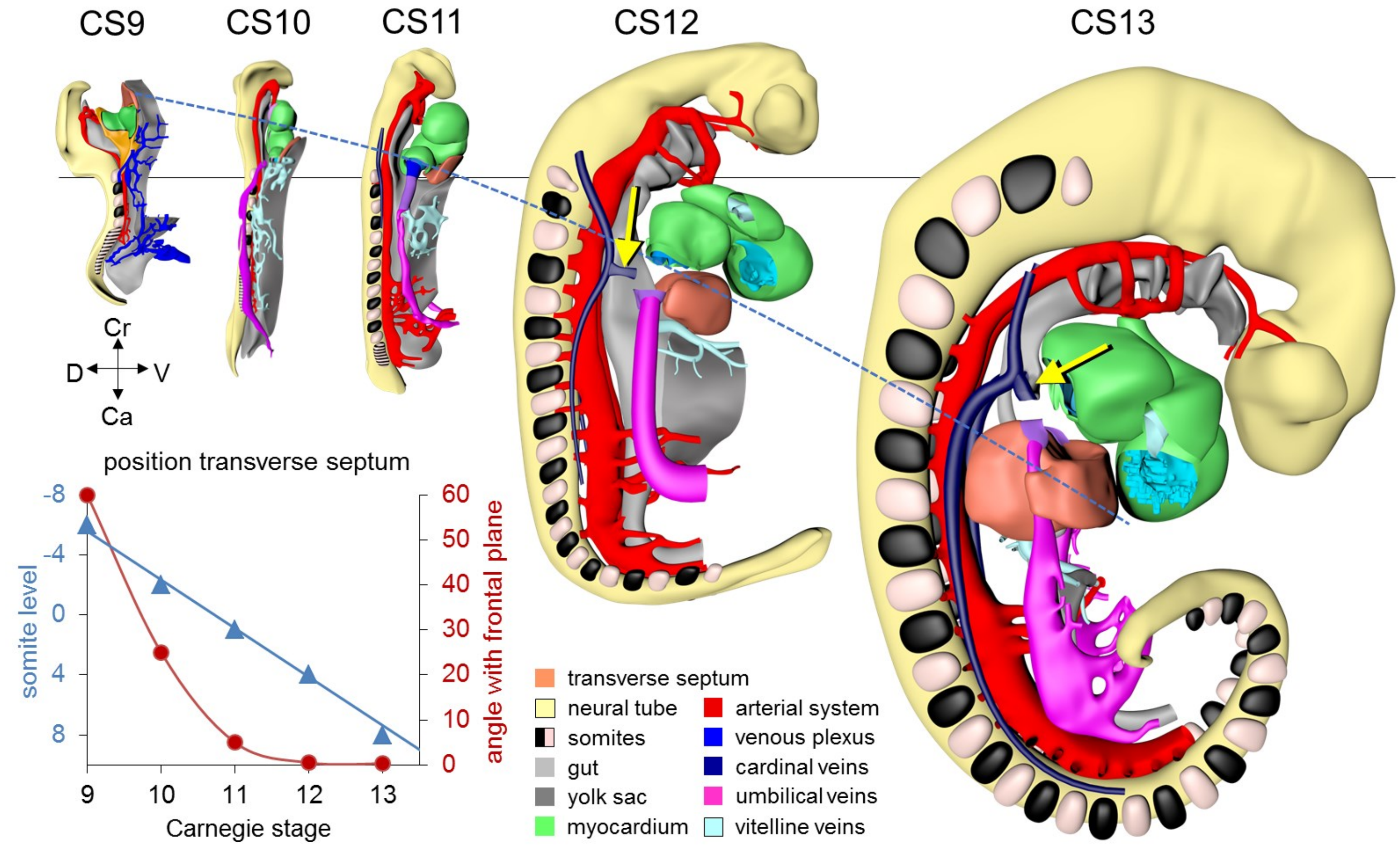

Supplemental Figure 14. Pictorial timeline of the 'descent" of the transverse septum. Between CS9 and CS13, the transverse septum and, along with it, the heart acquires a progressively more caudal position relative to the body axis due to growth of dorsal structures, such as the neural tube. The horizontal line shows how we aligned all embryos on the position of the first somite. The second, broken, line passes through the middle of the transverse septum. We partially removed some organs to better visualize the septum. The curvature of this line reflects the increasing size of the somites. We determined the position of the transverse septum by placing a line through the center of the septum perpendicular to the curvature of the body axis. This position relative to the first somite is then expressed in the number of somites, a negative number indicating that the septum is situated cranially to the first somite. The blue triangular symbols in the graph (each dot represents an embryo) show that the position of the septum "moves" caudally at ~3 somite lengths per developmental stage, while the red circular symbols show that the position of the septum rotates in a frontal plane across ~60° between CS9 and CS11. The direction of the common cardinal vein (yellow arrow) changes from being oriented transversely at CS12 to achieving a frontal position at CS13, this change also reflecting the descent of the heart. All images are also available as preset views in the corresponding 3D-PDFs. (hyperlink: 10.6084/m9.figshare.19102616)

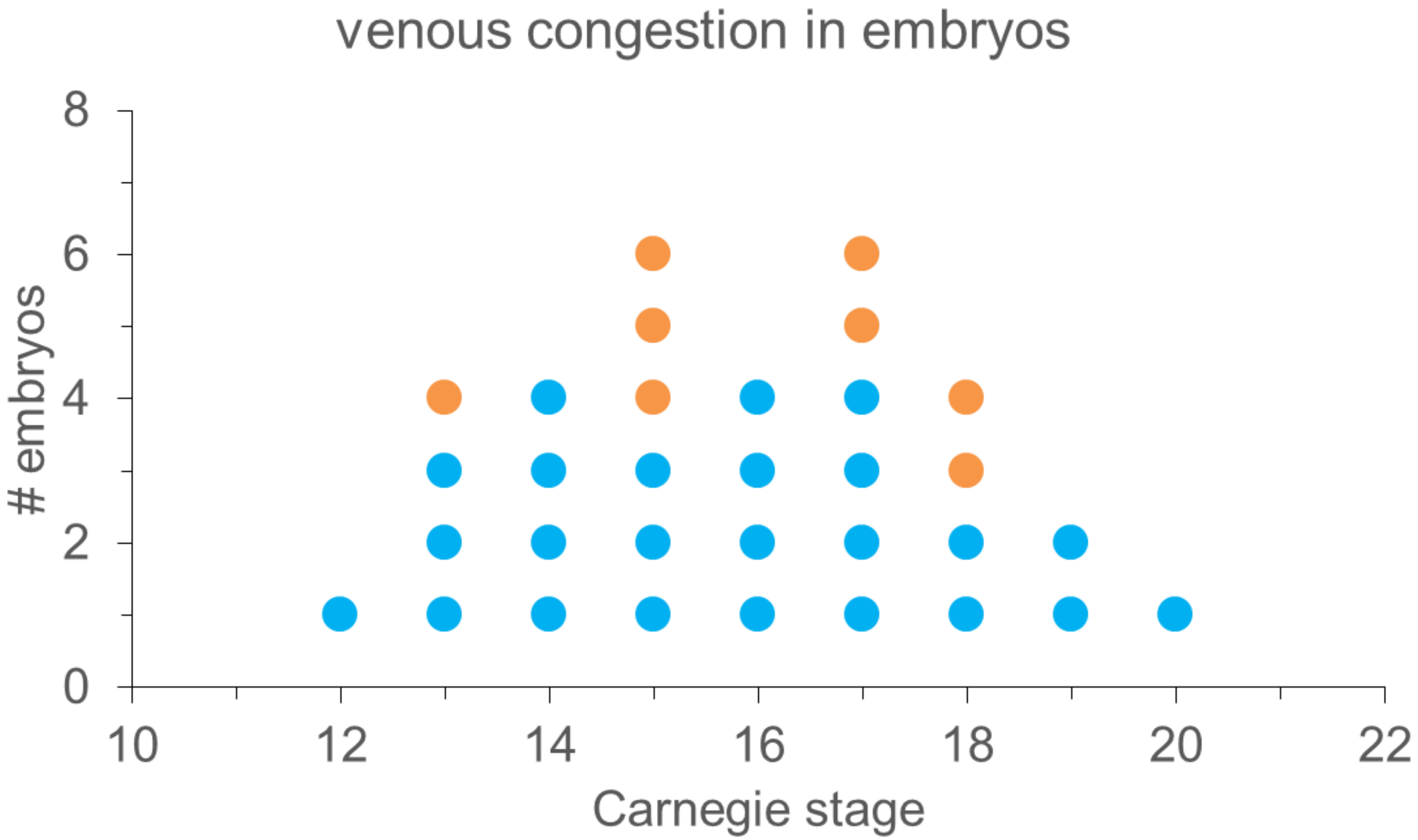

Supplemental Figure 15. Prevalence of venous congestion in 32 well-preserved digitized human embryos of the Blechschmidt collection. The authors were not involved in the selection of the embryos for scanning. (hyperlink: 10.6084/m9.figshare.17143970)

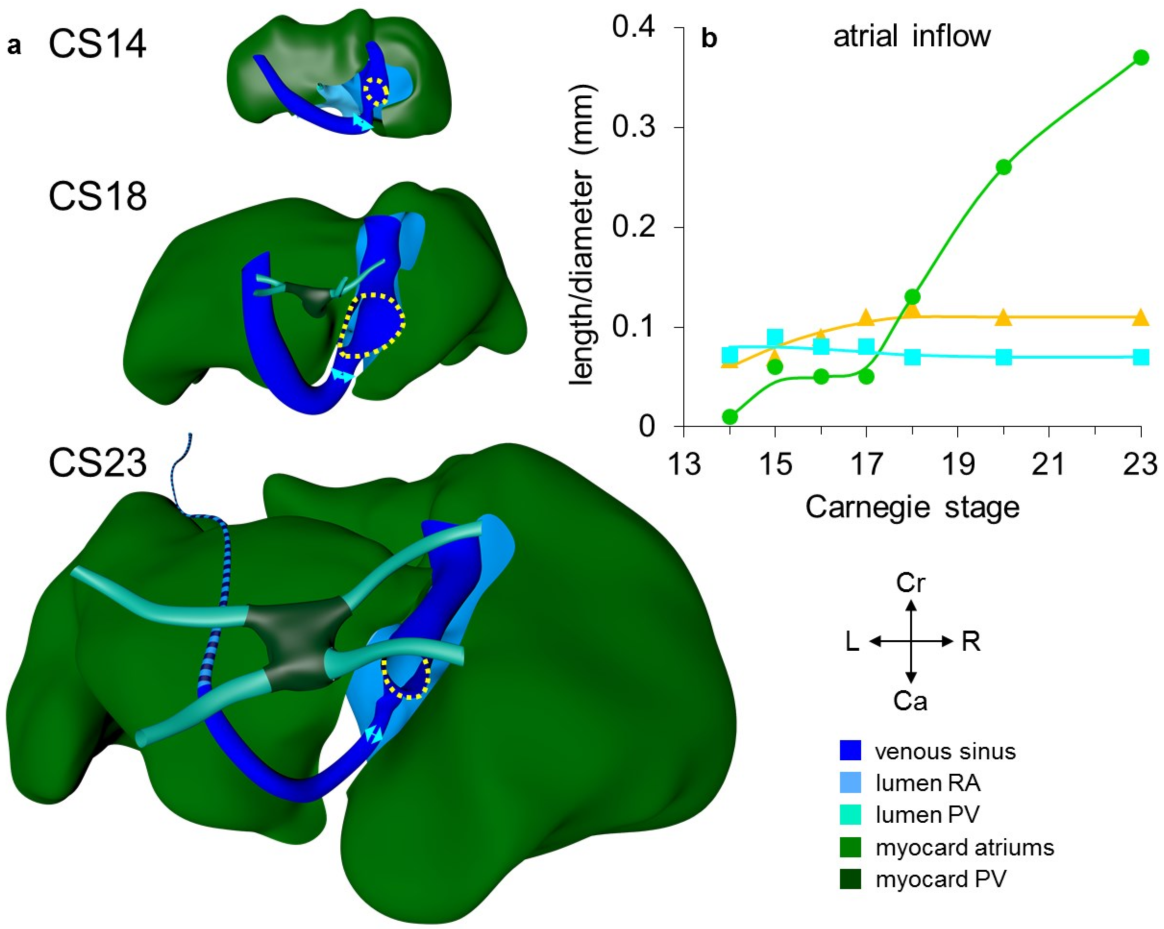

Supplemental Figure 16. Pictorial timeline of the developing pulmonary veins and coronary sinus. Each dot in the graph (panel b) represents a single embryo. The pulmonary vein acquires a lumen at CS14, while its stem begins to myocardialize at CS18 (panel a). After the initial increase in diameter of the pulmonary vein (green circular symbols, panel b), which reflects the appearance of its lumen, the diameter hardly changes up to CS17. Thereafter, the diameter of the pulmonary stem rapidly increases, whereas the axial length up to its first bifurcation remains constant (yellow triangular symbols). This growth pattern presages the absorption of the myocardialized part of the pulmonary vein in the wall the left atrium. Between CS14 and CS23, the diameter of the left sinus horn near its confluence with the right sinus horn remains constant (cyan square symbols in panel b and double-headed arrows in panel a), implying a gradual decrease in blood flow. Between CS21 and CS22, the distal portion of the left sinus horn and left superior caval vein begin to shrink and become the ligament of Marshall (hatched blue coding), while the proximal portion of the sinus horn becomes the coronary sinus. The yellow dashed ring (panel a) marks the position of the inferior caval vein. All images are also available as preset views in the corresponding 3D-PDFs.
(hyperlink: 10.6084/m9.figshare.19102622)

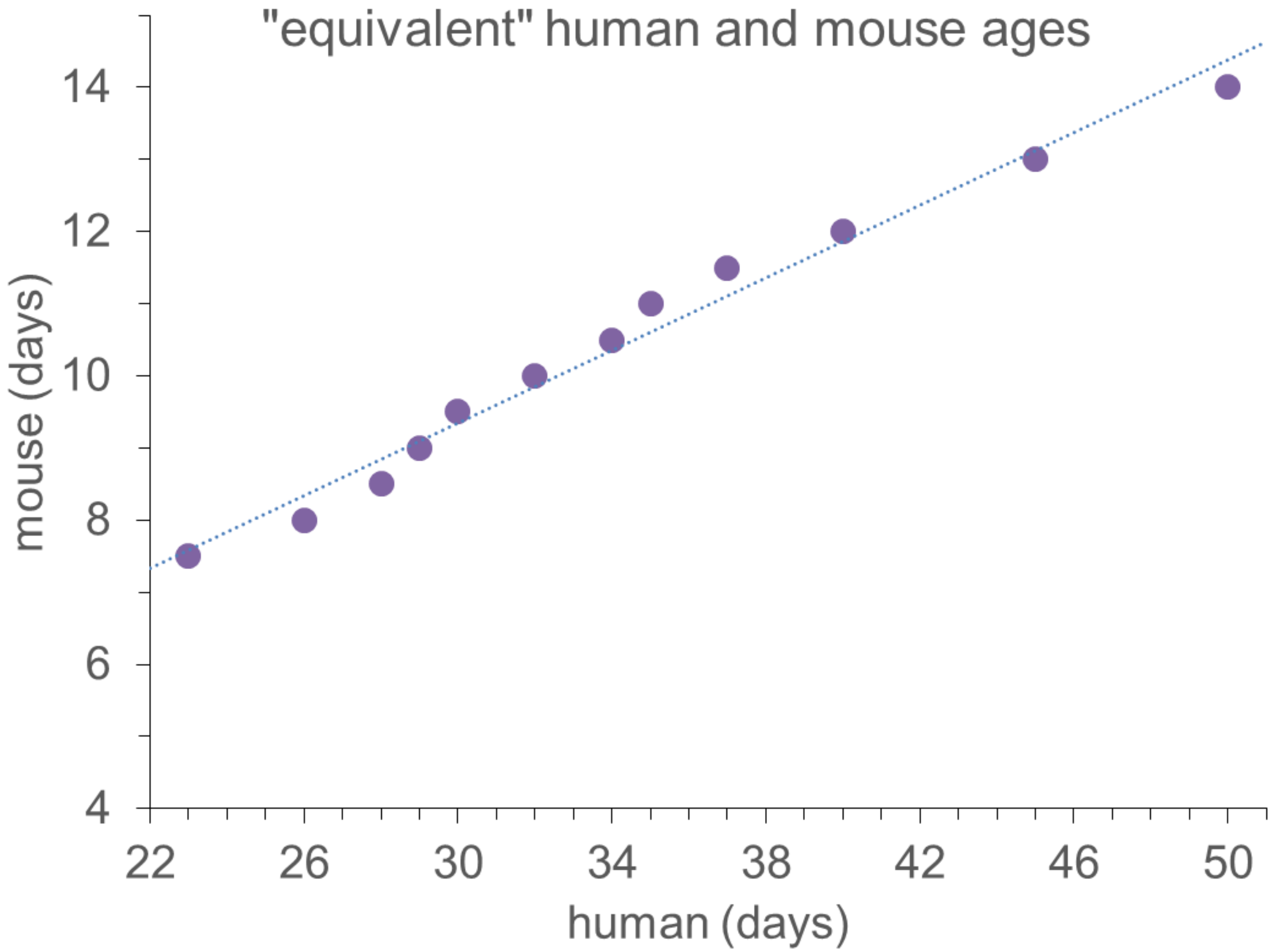

Supplemental Figure 17. Equivalent ages of human and mouse embryos. Similar Carnegie stages of development were recalculated to days of development after fertilization using Theiler's staging system for mouse embryos [7] and O'Rahilly's "proposed ages" for human embryos [1]. The regression coefficient (0.25) shows that 4 days of development in human embryos is equivalent to one day of development in mouse embryos throughout the period considered. To validate the correlation for the early times points, we staged our reconstructed early human hearts according to Le Garrec's system for early mouse hearts [9]. The CS9, CS10, and CS11 hearts correspond to their stages E8.5d, E8.5g, and E8.5j, respectively. These mouse embryos have 3-5, 7-8, and ≥11 somites, respectively [9], which corresponds to ~ED8, ~ED8.5, and ~ED9, respectively, in Theiler's staging system. Judging from the number of somites and the appearance of the ventricular wall, our CS12 human embryo should be comparable to an ~ED9.5 mouse embryo [7,10]. For discussion, see text. (hyperlink: 10.6084/m9.figshare.17144036)

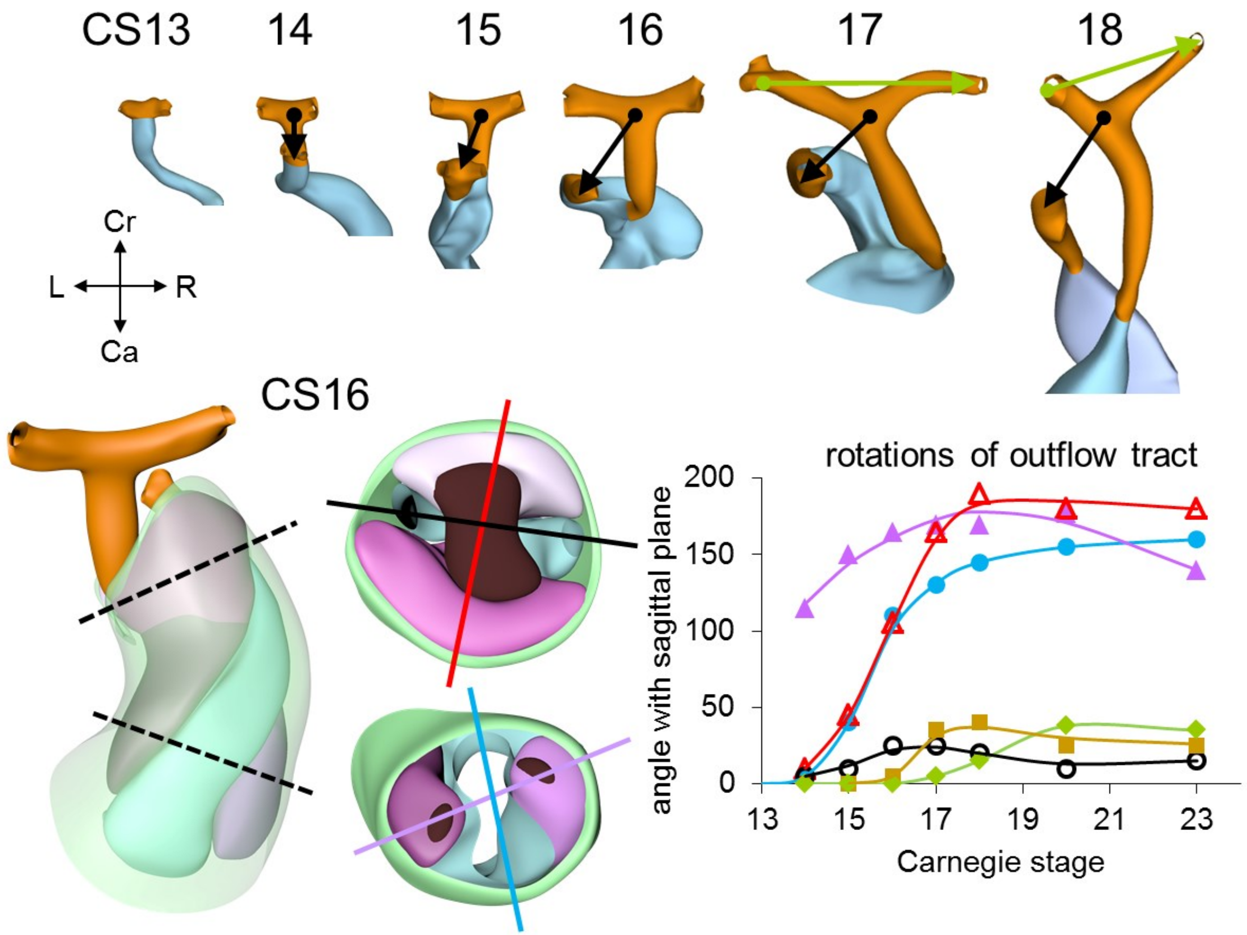

Supplemental Figure 18. Measurements to assess the degree of spiraling of the walls of the heart and arterial trunks. The upper panel shows caudal views of the lumens of the outflow tract and arterial trunks, and illustrates the protocol of the measurements shown in Figure 9. The black arrows show the rotation of the arterial trunks relative to each other between CS14 and CS18, while the green arrows reveal the asymmetric growth of the extrapericardial "horns" of the aortic trunk. The lower panel shows a ventral view of the outflow tract of a CS16 heart, with the myocardium rendered translucent. The dashed black lines indicate the position of the measurements. The degree of rotation of the subaortic and subpulmonary channels, the ridges, and the distally fused prongs of neural crest cells was determined perpendicular to the luminal axis of the outflow tract. The graph shown in Figure 9 is included for convenience. (hyperlink: 10.6084/m9.figshare.17144090)

A pictorial account of the human embryonic heart between 3.5 and 8 weeks of development. Hikspoors et al. 2021

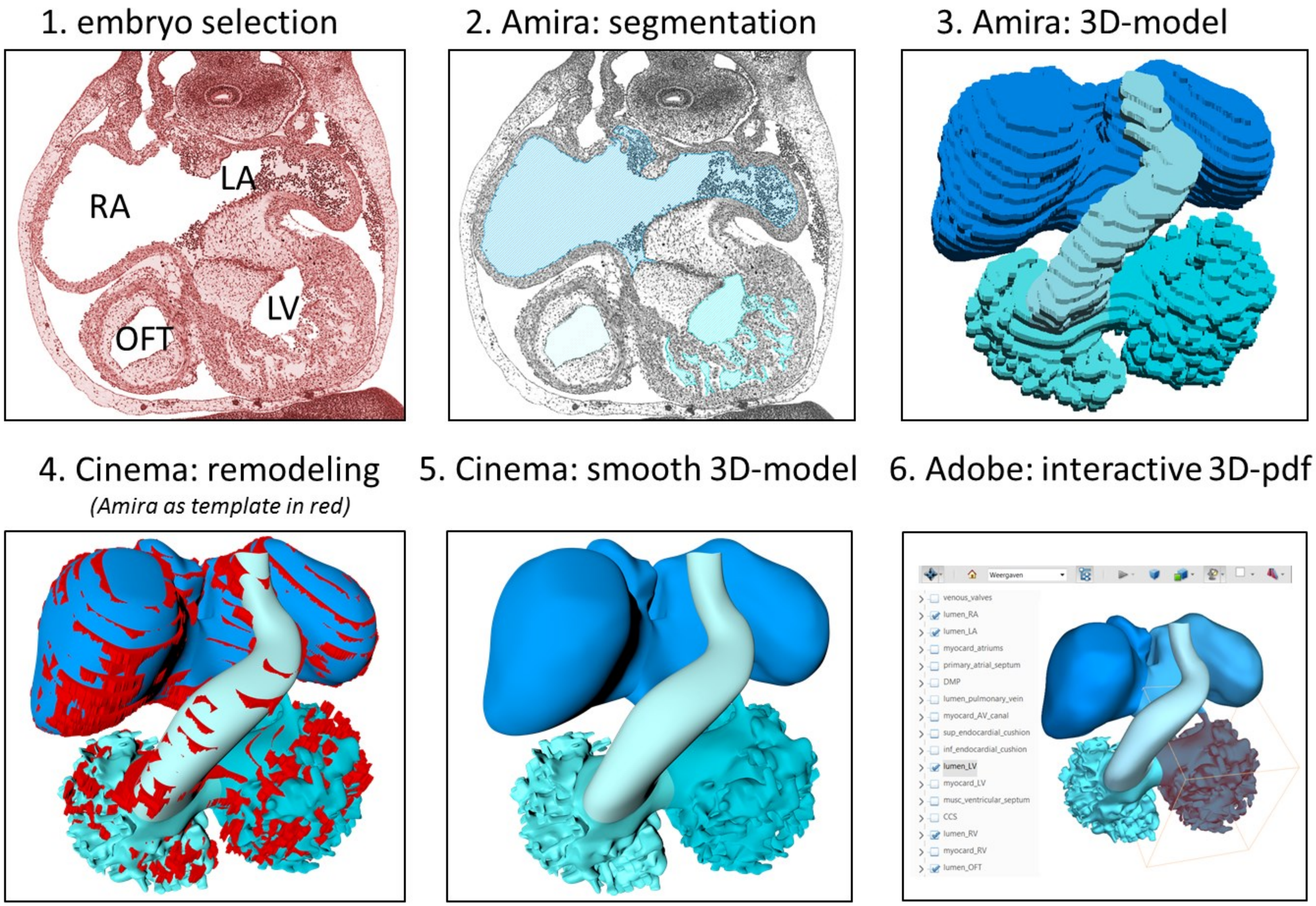

Supplemental Figure 19. Brief procedure of 3D reconstruction, analysis and rendering. (hyperlink: 10.6084/m9.figshare.17144114)

A pictorial account of the human embryonic heart between 3.5 and 8 weeks of development. Hikspoors et al. 2021

Supplementary References

1 O'Rahilly, R. & Muller, F. Developmental stages in human embryos: revised and new measurements. *Cells Tissues Organs* **192**, 73-84, doi:10.1159/000289817 (2010).

2 O'Rahilly, R. & Muller, F. *Developmental stages in human embryos, including a revision of Streeter's "horizons" and a survey of the Carnegie Collection*. Vol. 637 (1987).

3 Anderson, R. H. *et al.* Sequential segmental analysis of congenital heart disease. *Pediatr Cardiol* **5**, 281-287, doi:10.1007/BF02424973 (1984).

4 Manasek, F. J. Macromolecules of the extracellular compartment of embryonic and mature hearts. *Circ Res* **38**, 331-337 (1976).

5 Sizarov, A. *et al.* Formation of the building plan of the human heart: morphogenesis, growth, and differentiation. *Circulation* **123**, 1125-1135, doi:10.1161/CIRCULATIONAHA.110.980607 (2011).

6 Streeter, G. L. The development of the cranial and spinal nerves in the occipital region of the human embryo. *Am J Anat* **4**, 83-116 (1905).

7 Theiler, K. *The house mouse. Development and normal stages from fertilization to 4 weeks of age.*, (Springer-Verlag, 1972).

8 Hamburger, V. & Hamilton, H. L. A series of normal stages in the development of the chick embryo. *J Morphol* **88**, 49-92 (1951).

9 Le Garrec, J. F. *et al.* A predictive model of asymmetric morphogenesis from 3D reconstructions of mouse heart looping dynamics. *Elife* **6**, doi:10.7554/eLife.28951 (2017).

10 Tam, P. P. The control of somitogenesis in mouse embryos. *J Embryol Exp Morphol* **65 Suppl**, 103-128 (1981).
