# Supplementary material for: A pictorial account of the human embryonic heart between 3.5 and 8 weeks of development
Source: Commun Biol. 2022 Mar 11;5:226. doi: 10.1038/s42003-022-03153-x (PMC8917235; doi:10.1038/s42003-022-03153-x)
Supplement: Supplementary file 3 — Reporting Summary [file 42003_2022_3153_MOESM3_ESM.pdf]

nature research

Corresponding author(s): DBPR Manuscript COMMSBIO-20-3607

Last updated by author(s): 28-12-2020

Reporting Summary

Nature Research wishes to improve the reproducibility of the work that we publish. This form provides structure for consistency and transparency in reporting. For further information on Nature Research policies, see our Editorial Policies and the Editorial Policy Checklist.

Statistics

For all statistical analyses, confirm that the following items are present in the figure legend, table legend, main text, or Methods section.

n/a	Confirmed	
☒	☐	The exact sample size (n) for each experimental group/condition, given as a discrete number and unit of measurement
☒	☐	A statement on whether measurements were taken from distinct samples or whether the same sample was measured repeatedly
☒	☐	The statistical test(s) used AND whether they are one- or two-sided<br>*Only common tests should be described solely by name; describe more complex techniques in the Methods section.*
☒	☐	A description of all covariates tested
☒	☐	A description of any assumptions or corrections, such as tests of normality and adjustment for multiple comparisons
☒	☐	A full description of the statistical parameters including central tendency (e.g. means) or other basic estimates (e.g. regression coefficient) AND variation (e.g. standard deviation) or associated estimates of uncertainty (e.g. confidence intervals)
☒	☐	For null hypothesis testing, the test statistic (e.g. F, t, r) with confidence intervals, effect sizes, degrees of freedom and P value noted<br>*Give P values as exact values whenever suitable.*
☒	☐	For Bayesian analysis, information on the choice of priors and Markov chain Monte Carlo settings
☒	☐	For hierarchical and complex designs, identification of the appropriate level for tests and full reporting of outcomes
☒	☐	Estimates of effect sizes (e.g. Cohen's d, Pearson's r), indicating how they were calculated

Our web collection on statistics for biologists contains articles on many of the points above.

Software and code

Policy information about availability of computer code

Data collection	Digitized images of staged human embryos were obtained from the Digitally Reproduced Embryonic Morphology (DREM) project (Dr John Cork; Cell Biology & Anatomy, LSU Health Sciences Center, New Orleans; https://www.ehd.org/virtual-humanembryo/about.php, http://virtualhumanembryo.lsuhsc.edu).<br>The Department Anatomy & Embryology, Maastricht University, the Netherlands, holds licenses for reconstruction software Amira 3D and remodelling software Cinema 4D.
Data analysis	Amira 3D, Cinema 4D and microscoft Excel software were used for analysis of data

For manuscripts utilizing custom algorithms or software that are central to the research but not yet described in published literature, software must be made available to editors and reviewers. We strongly encourage code deposition in a community repository (e.g. GitHub). See the Nature Research guidelines for submitting code & software for further information.

Data

Policy information about availability of data

All manuscripts must include a data availability statement. This statement should provide the following information, where applicable:

- Accession codes, unique identifiers, or web links for publicly available datasets
- A list of figures that have associated raw data
- A description of any restrictions on data availability

Data availability:
- The accession code of the DREM embryo database is present in the Material & Methods section
- All Figures were prepared using the 3D-PDFs that are supplied as Supplemental Figures
- There are no restrictions on data availability. We confirm hereby that the web links to our Supplemental data and interactive 3D-PDFs are publicly available.

Field-specific reporting

Please select the one below that is the best fit for your research. If you are not sure, read the appropriate sections before making your selection.

☒ Life sciences ☐ Behavioural & social sciences ☐ Ecological, evolutionary & environmental sciences

For a reference copy of the document with all sections, see nature.com/documents/nr-reporting-summary-flat.pdf

Life sciences study design

All studies must disclose on these points even when the disclosure is negative.

Sample size	Staged human embryos were obtained from the Digitally Reproduced Embryonic Morphology (DREM) project, which are part of the Carnegie collection, Washington D.C., USA. Supplemental Table 1 shows the identifying Carnegie number of each embryo used. In addition to the reconstructed and modeled embryos, we also studied the immunohistochemically stained sections of human embryonic hearts collected and produced by Viragh and Wessels, Sizarov and Ya (for references, see Material & Methods section).
Data exclusions	No data was excluded from the analyses
Replication	No replication studies were carried out. Segmentation of structures was based on histological criteria and supported by inspection of immunohistochemically stained sections of human embryonic hearts.
Randomization	The DREM embryos were selected, independently of the present study, by Drs Gasser and Cork as representative for the indicated developmental stage. We focused on differences between stages, using a literature review to underscore the conclusion.
Blinding	No blinding was included. Blinding would be difficult since only one embryo per stage was reconstructed. Furthermore, embryos have a very characteristic shape. The emphasis of the study is on visualization of stage-specific features and their stage-dependent change in shape and size. These changes were highlighted in "timelines". The timelines included quantification of some of the changes, with each stage represented by a single value. Based on our experience, changes between stages far exceed variation between same-stage embryos.

Reporting for specific materials, systems and methods

We require information from authors about some types of materials, experimental systems and methods used in many studies. Here, indicate whether each material, system or method listed is relevant to your study. If you are not sure if a list item applies to your research, read the appropriate section before selecting a response.

Materials & experimental systems

n/a	Involved in the study	
☒	☐	Antibodies
☒	☐	Eukaryotic cell lines
☒	☐	Palaeontology and archaeology
☒	☐	Animals and other organisms
☒	☐	Human research participants
☒	☐	Clinical data
☒	☐	Dual use research of concern

Methods

n/a	Involved in the study	
☒	☐	ChIP-seq
☒	☐	Flow cytometry
☒	☐	MRI-based neuroimaging
